# Clinical frailty scale predicts outcomes following total joint arthroplasty

**DOI:** 10.1186/s42836-024-00294-8

**Published:** 2025-03-03

**Authors:** Benjamin J. Wall, Matthias Wittauer, Karlia Dillon, Hannah Seymour, Piers J. Yates, Christopher W. Jones

**Affiliations:** 1 Department of Orthopaedic Surgery, Fiona Stanley Fremantle Hospitals Group, South Metropolitan Health Service, Perth, 6150 Australia; 2 The Orthopaedic Research Foundation of Western Australia (ORFWA), Perth, 6150 Australia; 3 University of Basel, Basel, 4001 Switzerland; 4 Department of Anaesthesia, Pain and Perioperative Medicine, Fiona Stanley Fremantle Hospitals Group, South Metropolitan Health Service, Perth, 6150 Australia; 5 Department of Geriatric Medicine, Fiona Stanley Fremantle Hospitals Group, South Metropolitan Health Service, Perth, 6150 Australia; 6 University of Western Australia, Perth, 6009 Australia; 7 Curtin University, Perth, 6120 Australia

**Keywords:** Arthroplasty outcomes, TKA, THA, Frailty, Preoperative assessment, Length of stay, Complications, Patient-reported outcome measures

## Abstract

**Background:**

As the population ages, the number of total joint arthroplasty (TJA) performed is rising, making early identification of patients at risk for adverse events essential to improving care and reducing healthcare costs. The aim of this study was to evaluate the association between Clinical Frailty Scale (CFS) and postoperative outcomes in elective total hip arthroplasty (THA) and total knee arthroplasty (TKA).

**Methods:**

We conducted a retrospective study of prospectively collected data regarding 328 TKAs and 294 THAs at a single institution from February 2019 to February 2020. Patient demographic data were harvested, and the preoperative CFS scores were calculated for all patients and analyzed to identify their associations with the length of stay (LOS), the need for admission to an inpatient rehabilitation unit (IPRU), postoperative complications and patient-reported outcome measures (PROMs).

**Results:**

Robust patients (CFS < 3) had a significantly shorter LOS than their non-robust (CFS > 3) counterparts in both the TKA and THA groups (3.7 vs. 5.2 days, *P* < 0.001, and 3.8 vs. 5.8 days, *P* < 0.001, respectively). IPRU admission rates were significantly higher in non-robust than in robust patients. Specifically, none of the robust TKA patients required IPRU admission, whereas up to 39 non-robust patients (11.9%) did (*P* < 0.001). Similarly, for THA, 9 robust (5.7%) and 30 non-robust (21.9%) patients were admitted to an IPRU (*P* < 0.001). Non-robust patients had a significantly higher complication rate for both THA (11.0% vs. 6.4%, *P* = 0.03) and TKA (8.7% vs. 2.6%, *P* = 0.11). Both cohorts showed significant improvements in PROMs post-surgery, with non-robust patients experiencing greater relative gains than robust patients.

**Conclusions:**

CFS is a strong predictor of the hospital length of stay, IPRU admission, and complication rates following TJA. This study also highlighted the link between frailty and PROMs in joint replacement patients. The CFS may be a valuable tool in the preoperative assessment of elective THA and TKA.

## Background

More than half of all elective surgeries are performed in older adults (aged over 65 years), with total joint arthroplasty (TJA) being the most frequently performed procedures in this age group [1–3]. In 2024, the Australian Orthopaedic Association National Joint Replacement Registry (AOANJRR) recorded 58,529 total hip arthroplasty (THA) procedures performed in Australia, representing a 94.0% increase since 2006 [4]. Similarly, the annual incidence of total knee arthroplasty (TKA) in 2024 reached 78,125, reflecting a 145.1% increase over the same period. This significant increase can be attributed to the expansion of indication criteria for TJA, advancements in surgical techniques, rising obesity rates and the growing average age of the general population [5]. By 2030, the annual incidences of THA and TKA in Australia are projected to exceed 79,000 and 161,000 respectively [4].

As the population continues to age, this trend is expected to increase not only the number of TJA procedures but also the prevalence of comorbidities, which could further raise complication rates in arthroplasty patients [6]. TJA patients at higher risk for complications require greater resources and experience higher readmission rates, leading to increased costs [7, 8]. Early identification of at-risk patients is crucial for improving care quality in older adults undergoing TJA. Preoperative risk prediction allows for the optimization of modifiable factors and more effective resource allocation, potentially enhancing outcomes and lowering healthcare costs [9].

While comorbidity scores have been widely used to identify high-risk patients [10, 11], frailty has emerged as a stronger predictor of postoperative outcomes than both comorbidity scores and some procedure-specific risk models [12–15]. Frailty, a multifactorial syndrome characterized by decreased physiological reserve and reduced capacity to respond to stressors such as surgery [12], is associated with significantly increased morbidity and mortality rates in the perioperative period [16–22].

Evaluating frailty involves assessing an individual’s physical and cognitive capacity to function in the context of their local and systemic disease [17]. The Clinical Frailty Scale (CFS) was first introduced by Rockwood et al. in 2005 [23] as a 7-point clinical judgement-based frailty scale, that is easily performed by surgeons, physicians, and nurses from different specialties and has since been expanded to a 9-point semi-quantitative scaling system to differentiate between levels of frailty on the basis of specific domains including function, comorbidity, and cognition, ranging from 1 (very fit) to 9 (terminally ill) [24]. The CFS score can be reliably assigned retrospectively and has been shown to be a valid diagnostic instrument to measure frailty in older hospitalized patients [25–27].

In patients undergoing orthopedic surgery, the CFS has been linked to prolonged length of stay (LOS) following TJA [28] and serves as a strong predictor of mortality in patients with surgically managed hip periprosthetic and proximal femur fractures [20, 21, 29]. Despite its proven predictive value, the association between frailty and postoperative outcomes in elective TJA remains underexplored.

The aim of this study was to examine the relationships between CFS and postoperative outcomes, including hospital LOS, the need for admission to an inpatient rehabilitation unit (IPRU), complication rates and patient-reported outcome measures (PROMs), following primary THA and TKA. We hypothesized that patients with a higher CFS score would have a longer LOS, a greater likelihood of IPRU admission, increased perioperative complications and lower overall PROMs.

## Methods

### Study design and patient selection

We conducted a retrospective observational study of prospectively collected data from a consecutive series of patients who presented at our elective arthroplasty center at a tertiary public hospital to undergo either primary total knee arthroplasty (TKA) or total hip arthroplasty (THA) between February 1, 2019, and February 31, 2020. Using an electronic medical record (EMR), a retrospective review was conducted to identify all elective primary THAs and TKAs performed during the time period. Data were collected from the EMRs and institutional database, including patient and operating room records, operation reports, discharge and clinic letters. A total of 622 patients were included in the final analysis.

### Data collection and outcome measures

All patients scheduled to undergo joint replacement surgery were routinely reviewed in both the orthopedic outpatient setting and in a multidisciplinary preadmission clinic. If a patient was identified as being at high risk for perioperative complications, they were evaluated in a high-risk preoperative anaesthetic clinic with input from allied health, anaesthetic and surgical teams. Patients were reviewed postoperatively in both the orthopedic and physiotherapy outpatient clinics, where PROMs were collected.

We extracted the following parameters:

1. Baseline demographics: age, sex and BMI.
2. Hospital length of stay (LOS).
3. Admission to an IPRU.
4. Perioperative complications: the number and type of perioperative complications were recorded. A medical complication was defined as a new medical problem arising during the hospital stay, whereas a surgical complication involved a problem related directly to the operation that required further intervention or surgery during the postoperative period. For patients with multiple complications, only the most severe complication was recorded.
5. PROMs collected routinely at 6 weeks and 1 year postoperatively: Joint specific Osteoarthritis Outcome Scores (Hip disability and osteoarthritis outcome score [HOOS] or Knee Injury and Osteoarthritis Outcome Score [KOOS], depending on the joint operated on), Forgotten Joint Score (FJS), Oxford 12 score and EQ-5D-5LVAS were harvested.
6. CFS: Two assessors (B.W. and K.D.) retrospectively reviewed all patients’ EMRs to calculate frailty using the CFS. The CFS scoring is detailed in Table 1 [24]. CFS assessment was based on orthopedic and geriatric admission notes, which detailed cognitive function, medical comorbidities, admission residence, premorbid fitness/activity, mobility aids, and the level of services or support received at home or in residential care. The CFS score was calculated using preoperative data from a subset of the patients’ EMR, with blinding to PROMs, LOS, IPRU admission, and arthroplasty outcomes. In addition, patients were subdivided into two cohorts based on the CFS: a robust (CFS ≤ 3) and a non-robust (CFS > 3) subgroup, with a score of 4 or higher indicating frailty [24]. In case of discrepancy in CFS scoring, the EMR was reviewed independently by the senior author (C.J.) to reach a consensus.

**Table 1:** Detailed clinical frailty scale

Group	CFS Category	Type	Details
Robust (CFS ≤ 3)	1	Very fit	Individuals who are robust, active, and energetic, regularly exercising and among the fittest for their age
	2	Fit	Individuals without active disease symptoms but less fit than Category 1, often engaging in occasional or seasonal exercise
	3	Managing well	Individuals whose medical issues are well controlled but who are not regularly active beyond routine walking
Non-robust (CFS > 3)	4	Living with very mild frailty	People who are not dependent on others for daily help but have symptoms that limit activities, causing them to feel “slowed up” or tired during the day
	5	Living with mild frailty	Individuals who are characterized by more evident slowing, with need for assistance with high order instrumental activities of daily living such as finances, transportation, and heavy housework. They may also struggle with shopping, walking outside alone, meal preparation, and housework
	6	Living with moderate frailty	People who need help with all outside activities and maintaining the house, often having problems with stair-climbing, bathing, and may require minimal assistance with dressing
	7	Living with severe frailty	Individuals who are completely dependent for personal care, whether due to physical or cognitive issues, but who appear stable and are not at high risk of dying within approximately six months
	8	Living with very severe frailty	People who are completely dependent and nearing the end of life, unable to recover from even minor illnesses
	9	Terminally ill	Individuals with a life expectancy of less than six months, who are approaching the end of life and are not otherwise evidently frail

*CFS* clinical frailty scale

### Statistical methods

Statistical analysis was performed via the XLMiner Analysis Tool in Microsoft Excel and SPSS Statistics, version 28 (IBM Corp., Armonk, NY, USA). Categorical data are presented as percentages. Categorical covariates were calculated using chi-squared tests. Continuous variables are expressed as mean ± standard deviation (SD) and underwent one-way analysis of variance. Bivariate analyses were performed to test the associations between different frailty measures and the outcomes of interest (hospital LOS > 5 days, admission to the IPRU, PROMs at 6 weeks and 1 year). Ordinal logistic regression was used to test the association of CFS with complications.

This study received Institutional Review Board approval (GEKO No. 35557).

## Results

### Baseline demographics

The study recruited 622 patients, including 328 patients who underwent TKA and 294 patients who received THA. Table 2 presents the baseline patient demographic data.

**Table 2:** Baseline demographics

Group	Variable	Total	Robust cohort (CFS ≤ 3)	Non-robust cohort (CFS > 3)	*P*-value
TKA	Patients, *n* (%)	328 (100%)	76 (23.2%)	252 (76.8%)	
	Age in yrs, mean ± SD	67.5 ± 9.8	62.2 ± 7.3	69 ± 10	< 0.001
	Female sex, *n* (%)	184 (56.1%)	38 (50.0%)	146 (57.9%)	0.022
	BMI, mean ± SD	32.5 ± 6.3	30.6 ± 5.4	33.1 ± 6.4	0.002
THA	Patients, *n* (%)	294 (100%)	157 (53.4%)	137 (46.6%)	
	Age in yrs, mean ± SD	64.3 ± 13.5	60.1 ± 12.6	69.3 ± 18	< 0.001
	Female sex, *n* (%)	159 (54.1%)	76 (48.4%)	83 (60.6%)	0.034
	BMI, mean ± SD	29.3 ± 6.5	28.3 ± 6.0	30.6 ± 6.8	0.004

*TKA* total knee arthroplasty, *THA* total hip arthroplasty, *yrs* years, *BMI* body mass index, *SD* standard deviation

Baseline characteristics were similar between the THA and TKA groups with respect to mean age (64.3 vs. 67.7 years), mean BMI (29.3 vs. 32.5) and sex distribution (52.4% vs. 55.4% female). In the TKA group, 76.5% of patients were classified as non-robust (CFS > 3) whereas in the THA group, 46.6% of patients fell into the same category. Patients in the non-robust cohort in both the THA and TKA groups were significantly older, presented a higher BMI and included a greater proportion of women than patients in the robust cohort. Table 3 shows a detailed overview of the CFS score assigned to the patient population.

**Table 3:** Clinical frailty scale score assigned to patients’ population

Group	Clinical Frailty Scale	TKA cohort, *n* (%)	THA cohort, *n* (%)	Type
Robust (CFS ≤ 3)	1	0	9 (3.1%)	Very fit
	2	1 (0.3%)	44 (15.0%)	Fit
	3	75 (22.9%)	104 (35.4%)	Managing well
Total robust		76 (23.2%)	157 (53.4%)	
Non-robust (CFS > 3)	4	179 (54.6%)	92 (31.3%)	Living with very mild frailty
	5	64 (19.5%)	39 (13.3%)	Living with mild frailty
	6	9 (2.7%)	6 (2.0%)	Living with moderate frailty
	7	0	0	Living with severe frailty
	8	0	0	Living with very severe frailty
	9	0	0	Terminally ill
Total non-robust		252 (76.8%)	137 (46.6%)	
Total		328 (100%)	294 (100%)	

*TKA* total knee arthroplasty, *THA* total hip arthroplasty

### Length of stay

Patients classified as robust had a significantly shorter mean length of stay (LOS) than non-robust patients in both the TKA and THA group (3.7 vs. 5.2 days, *P* < 0.001 and 3.8 vs. 5.8 days, *P* < 0.001, respectively) (Table 3). Each incremental increase in CFS score was associated with a 1.4-day increase in LOS (*P* < 0.001).

### IPRU admission

A total of 39 non-robust TKA patients (11.9%) were transferred to an IPRU, whereas none of the robust patients required IPRU admission (*P* ≤ 0.001). Among THA patients, a total of 39 (13.3%) were admitted to an IPRU, including 9 (5.7%) classified as robust and 30 (21.9%) as non-robust (*P* ≤ 0.001) (Table 4). Overall, each incremental increase in CFS score was associated with a 27% greater likelihood of requiring admission to an IPRU (*P* ≤ 0.001).

**Table 4:** Length of stay and IPRU admission rates

Group	Variable	Total	Robust cohort (CFS ≤ 3)	Non-robust cohort (CFS > 3)	*P*-value
TKA	Patients, *n*	328	76	252	
	Length of stay				< 0.001
	1–5 days, *n* (%)	263 (80.2%)	70 (92.1%)	193 (76.6%)	
	> 5 days, *n* (%)	65 (19.8%)	6 (7.9%)	59 (23.4%)	
	IPRU admission				< 0.001
	Yes, *n* (%)	39 (11.9%)	0	39	
	No, *n* (%)	289 (88.1%)	76	213	
THA	Patients, *n*	294	157	137	
	Length of stay				< 0.001
	1–5 days, *n* (%)	235 (79.9%)	144 (91.7%)	91 (66.4%)	
	> 5 days, *n* (%)	59 (20.1%)	13 (8.3%)	46 (33.6%)	
	IPRU admission				< 0.001
	Yes, *n* (%)	39 (13.3%)	9 (5.7%)	30 (21.9%)	
	No, *n* (%)	255 (86.7%)	148 (94.3%)	107 (78.1%)	

*IPRU* inpatient rehabilitation unit, *TKA* total knee arthroplasty, *THA* total hip arthroplasty

### Complication rates

Non-robust THA patients had a higher overall complication rate (11.0%) than the robust patients (6.4%) (*P* = 0.03). A similar trend was observed in TKA patients, with 8.7% of non-robust patients developing complications compared to 2.6% of robust patients, though this difference was not statistically significant (Table 5).

**Table 5:** Rates of complications

Group	Variable	Total	Robust cohort (CFS ≤ 3)	Non-robust cohort (CFS > 3)	Odds ratio	*P*-value
TKA	Patients, *n*	328	76	252		
	Complications, *n* (%)	24 (7.3%)	2 (2.6%)	22 (8.7%)	3.5	0.11
THA	Patients, *n*	294	157	137		
	Complications, *n* (%)	25 (8.5%)	10 (6.4%)	19 (11.0%)	2.4	0.03

*TKA* total knee arthroplasty, *THA* total hip arthroplasty

Table 6 presents an overview of complications, which included dislocations, wound healing problems, periprosthetic joint infections, fractures, venous thrombotic events, myocardial infarctions, urinary tract infections, delirium and unexpected intensive care unit (ICU) transfer. No patients died during their hospital stay.

**Table 6:** Overview of perioperative complications

	**TKA**			**THA**		
**Variable**	**Total**	**Robust cohort** **(CFS ≤ 3)**	**Non-robust cohort** **(CFS > 3)**	**Total**	**Robust cohort** **(CFS ≤ 3)**	**Non-robust cohort** **(CFS > 3)**
Patients, *n*	328	76	252	294	157	137
Total complications, *n* (%)	24 (7.3%)	2 (2.6%)	22 (8.7%)	25 (8.5%)	10 (6.4%)	19 (11.0%)
Dislocation, *n*	0	0	0	8	6	2
Wound healing problems, *n*	3	1	2	3	3	0
Periprosthetic Joint Infection, *n*	3	0	3	6	1	5
Fracture, *n*	0	0	0	3	0	3
DVT, *n*	3	0	3	2	0	2
Pulmonary embolism, *n*	3	0	3	1	0	1
Myocardial infarction, *n*	1	0	1	0	0	0
Hospital acquired pneumonia, *n*	0	0	0	1	0	1
Urinary tract infection, *n*	1	0	1	0	0	0
Delirium	4	0	4	0	0	0
Transfer to ICU, *n*	6	1	5	1	0	1

*TKA* total knee arthroplasty, *THA* total hip arthroplasty, *DVT* deep vein thrombosis, *ICU* intensive care unit

### Patient-reported outcome measures (PROMs)

A comparison of PROMs over time between the robust and non-robust cohorts in both the TKA and THA groups is outlined in Table 7. Compared with robust patients, non-robust patients had significantly lower preoperative PROMs, including EQ-5D VAS, KOOS/HOOS, and Oxford 12 scores, in both the TKA and THA groups. However, these differences began to diminish by six weeks postoperatively, with both cohorts yielding similar PROMs by one year. At the six-week follow-up, the mean EQ-5D VAS score in the non-robust cohort surpassed that in the robust cohort (*P* = 0.04), whereas all other PROMs did not significantly differ at that time point.

**Table 7:** Patient reported outcome measures

	**TKA**			**THA**		
**Variable**	**Robust cohort** **(CFS ≤ 3)**	**Non-robust cohort (CFS > 3)**	***P*-value**	**Robust cohort** **(CFS ≤ 3)**	**Non-robust cohort** **(CFS > 3)**	***P*-value**
Patients, *n*	76	252		76	252	
EQ-5D VAS						
Preoperative, mean ± SD (*n*)	67.3 ± 20.2 (76)	59.4 ± 23.2 (240)	0.004	60.6 ± 23.6 (152)	52.6 ± 25.1 (128)	0.007
6 weeks, mean ± SD (*n*)	64.8 ± 24.6 (69)	71.5 ± 20.3 (215)	0.04	77.5 ± 20.6 (121)	73.2 ± 22 (96)	0.13
1 year, mean ± SD (*n*)	72.2 ± 25.7 (59)	73.4 ± 22.1 (208)	0.75	73.7 ± 25.4 (107)	78.4 ± 24.4 (98)	0.17
Forgotten Joint Score						
6 weeks, mean ± SD (*n*)	26.4 ± 25.5 (70)	32 ± 26.6 (214)	0.063	42.6 ± 30.8 (124)	46.6 ± 29.6 (98)	0.33
1 year, mean ± SD (*n*)	46.6 ± 32.6 (59)	50.4 ± 32.1 (209)	0.43	67.4 ± 31.5 (114)	62.4 ± 32.1 (90)	0.25
KOOS/HOOS 12 score						
Preoperative, mean ± SD (*n*)	37.1 ± 17.5 (76)	30.6 ± 15.3 (237)	0.004	35.9 ± 17.2 (152)	25.9 ± 15.5 (128)	< 0.001
6 weeks, mean ± SD (*n*)	57 6 ± 19.6 (70)	60.5 ± 18.5 (215)	0.27	70.8 ± 20.3 (123)	71.4 ± 19.9 (99)	0.8
1 year, mean ± SD (*n*)	73.7 ± 19.7 (59)	71.5 ± 22.0 (209)	0.47	84.7 ± 20.5 (115)	81.4 ± 20.9 (90)	0.25
Oxford 12 score						
Preoperative, mean ± SD (*n*)	20.8 ± 8.2 (76)	17.4 ± 7.9 (237)	0.002	20.2 ± 9.1 (153)	13.9 ± 7.3 (128)	< 0.001
6 weeks, mean ± SD (*n*)	27.5 ± 9.6 (70)	28 ± 8.7 (215)	0.69	32.4 ± 9.7 (124)	32.5 ± 8.9 (97)	0.92
1 year, mean ± SD (*n*)	36.3 ± 9.6 (59)	36.4 ± 9.0 (208)	0.98	41.4 ± 9.3 (115)	39 ± 9.1 (91)	0.06

*TKA* total knee arthroplasty, *THA* total hip arthroplasty, *EQ-5D VAS* EuroQol-5D visual analogue scale, *HOOS* Hip dysfunction and Osteoarthritis Outcome Score, *KOOS* Knee injury and Osteoarthritis Outcome Score, *SD* standard deviation

## Discussion

This study highlighted the potential of the CFS as a valuable tool for risk stratification in patients undergoing elective joint replacement surgery. Significant associations were identified between the level of frailty, as measured by the CFS, and key outcomes such as hospital length of stay, the need for inpatient rehabilitation, complication rates, and patient-reported outcome measures.

Our results demonstrated that non-robust patients (CFS > 3) had a longer overall LOS and higher rate of admission to IPRU than robust patients (CFS ≤ 3) following elective TJA. Each incremental increase in CFS score was associated with a 1.4-day increase in LOS and a 27% greater likelihood of requiring admission to an IPRU. These findings are consistent with those of Wang et al., who reported similar associations between CFS scores and discharge disposition in elective THA and TKA patients [28]. In their study, 17.5% of robust patients (CFS 1–3) had an LOS of more than five days, compared to 48.9% in non-robust patients (CFS 4–9). IPRU admission was also more frequent in non-robust patients (27.7%) than in robust patients (10%). Although Wang et al.’s study had several limitations, including a small sample size and reliance on patient self-assessment for the CFS scoring, its results mirror our findings of a longer LOS and higher IPRU admission rates for non-robust patients. In our study, the difference was particularly apparent within the TKA group where no patient with a CFS ≤ 3 was admitted to an IPRU. Even though variations in IPRU admission rates across hospitals are likely related to differing discharge and rehabilitation criteria, our findings reinforce the key role of frailty in predicting both LOS and the need for rehabilitation in the setting of elective TJA, a relationship already well established in other medical specialties [30].

Complication rates increased proportionally with higher CFS scores, with a statistically significant difference observed in THA patients. Despite having a lower proportion of non-robust patients (46.6%) than the TKA group (76.8%), the THA group had higher complication rates, longer LOS, and more frequent IPRU admissions. This might be ascribed to the higher overall complication, readmission, and reoperation rates typically associated with THA compared to TKA [31, 32]. These results strengthen the existing consensus on the relationship between frailty and surgical complication rates [17, 20, 28, 33].

Furthermore, our study provided valuable insights into the relationship between frailty and patient-reported outcome measures (PROMs) following total joint arthroplasty (TJA). For elective TJA, PROMs are useful in evaluating the success and overall patient benefit of orthopedic procedures, with increasing emphasis placed on joint function and quality of life [34]. Both the robust and non-robust cohorts in the TKA and THA groups demonstrated significant improvements in PROMs following joint replacement surgery. Clinical osteoarthritis is strongly associated with frailty and pre-frailty in older adults [35], and joint replacement is an effective intervention to improve frailty as well as quality of life in individuals with hip and knee osteoarthritis. After surgery, pre-frail individuals often regain robustness and frail patients frequently no longer meet the criteria for frailty [36]. Given that frailty significantly impacts quality of life [37, 38], addressing frailty through joint replacement can lead to substantial improvements in patient well-being. Although possible changes in CFS scores postoperatively were not assessed in this study, our results underscore the significant positive effect of TJA on quality of life in all patients with osteoarthritis, irrespective of their frailty status.

Our findings demonstrated that non-robust patients experienced greater relative improvements in PROMs postoperatively than robust patients. At six weeks post-surgery, the mean EQ-5D VAS score in the non-robust group even surpassed that of the robust cohort, despite the non-robust group starting with a lower baseline score. These findings suggest that the detrimental impact of osteoarthritis on quality of life is more pronounced in frail patients, and joint replacement might offer a more significant improvement in this group than in robust patients. Notably, the positive impact of joint replacement surgery on PROMs in non-robust patients was not compromised by the higher complication rates observed in this cohort.

In recent years, advanced data analysis tools, such as machine learning algorithms, have gained popularity for analyzing the impact of multiple medical comorbidities on postoperative outcomes [39–42]. Artificial intelligence (AI) models have shown the greatest accuracy in predicting postoperative complications, pain, and patient-reported outcomes, although they are less reliable in forecasting hospital readmission and reoperation [42, 43]. However, unlike these advanced tools, the CFS is simple, widely accessible, and easy to implement. This renders it particularly valuable for hospitals with limited resources and for surgeons in private practice who may not have access to advanced data analytics. By integrating the CFS into the routine preoperative assessment for TJA patients, frail individuals can be identified early, allowing for targeted preoperative optimization and efficient resource allocation. This may involve collaboration with geriatricians, general practitioners and physical therapists to improve mobility, function and overall health, potentially enhancing postoperative outcomes and identifying patients who may benefit from early admission to an IPRU.

This study has several limitations that should be considered. The retrospective application of the CFS, based on electronic medical records detailing patients' medical histories and functional status prior to admission, is a notable limitation, even though its retrospective application has been validated [25, 26]. Future research could benefit from recording the CFS score at admission to improve accuracy. Additionally, the study suffered from a relatively high dropout rate for PROM follow-up, with only approximately 70% of patients providing PROMs at the one-year mark. This dropout rate is largely attributable to disruptions in the local healthcare system due to the COVID-19 pandemic. Moreover, complication rates may be underestimated since complications were only recorded for patients who returned to our institution for subsequent treatment. Finally, the study did not evaluate the correlation between preoperative arthritis severity and its potential impact on patient frailty, nor did it assess postoperative CFS scores, which might be influenced by improvements in mobility and function following TJA.

## Conclusions

The results of our study revealed that the CFS is a strong predictor of hospital LOS, IPRU admission and complication rates following TJA. Additionally, this study demonstrated an association between frailty and PROMs in patients undergoing joint replacement. Integrating the CFS, a simple, widely accessible, and easy-to-implement tool, in the preoperative assessment of older adult patients in orthopedic clinics could facilitate the early identification of at-risk individuals. This would enable targeted perioperative medical and functional optimization, potentially reducing complication rates, and improving overall patient care, while alleviating the financial burden on the healthcare system.

## Data Availability

The datasets used and/or analyzed during the current study are available from the corresponding author upon reasonable request.

## Abbreviations

AI: Artificial intelligence
AOANJRR: Australian Orthopaedic Association National Joint Replacement Registry
BMI: Body mass index
CFS: Clinical Frailty Scale
DVT: Deep vein thrombosis
EMR: Electronic medical records
EQ-5D VAS: EuroQol-5D visual analogue scale
FJS: Forgotten Joint Score
HOOS: Hip Dysfunction and Osteoarthritis Outcome Score
ICU: Intensive care unit
IPRU: Inpatient rehabilitation unit
KOOS: Knee Injury and Osteoarthritis Outcome Score
LOS: Length of stay
PROMS: Patient-reported outcome measures
THA: Total hip arthroplasty
TJA: Total joint arthroplasty
TKA: Total knee arthroplasty
